# Changes in clozapine dose and concomitant medication - a 10-year comparative study

**DOI:** 10.1192/j.eurpsy.2024.450

**Published:** 2024-08-27

**Authors:** A. V. Popa, P. Petric, A. Teodorescu, P. I. Ifteni

**Affiliations:** ^1^Transilvania University of Brasov; ^2^Clinical Hospital of Psychiatry and Neurology of Brasov, Brasov, Romania

## Abstract

**Introduction:**

Clozapine is an atypical antipsychotic approved for treatment-resistant schizophrenia. Although effective, possible side effects make its underutilization still a current problem. The type of titration and dosages used differ worldwide.

**Objectives:**

To asses doses of clozapine and concomitant medications used in schizophrenia during 2012-2013 versus 2022-2023.

**Methods:**

A retrospective observational study analysing clozapine doses and concomitant treatment used in schizophrenia from 2012-2013 compared to 2022-2023. Data were collected from the medical charts of patients admitted to the Clinical Hospital of Psychiatry and Neurology Brasov, Romania, during 2012-2013 and 2022-2023.

**Results:**

In the total of 570 patients who were admitted in 2012-2013 with a diagnosis of paranoid schizophrenia, 69 (12,10%) of them were treated with clozapine.Of the 69 cases, 53,62% patients were females, mean age was 40,95 years (SD = ±10,32), with an average of onset age 23,17 (SD=±6,21). The average length of stay for hospitalization was 24,97 days (SD= ±12,65). The mean clozapine dose was 393,47 ((SD= ±183,69), with a minimum dose of 100mg/day and a maximum dose of 800mg/day. 37,68% of patients received concomitant treatment with benzodiazepines, mood stabilisers or sedative-hypnotic drugs. None of the patients received concomitant treatment with another antipsychotic.Among the total of 356 patients admitted with the diagnosis of paranoid schizophrenia during the 2022-2023 period, 72 (20,22%) of the patients were treated with clozapine. 72,22% patients were females, mean age was 49,12 years (SD = ±11,16), with an average of onset age 25,04 (SD=±6,40). The average length of stay for hospitalization was 18,58 days (SD= ±13,78). The mean clozapine dose was 275,34 (SD= ±146,7), with a minimum dose of 25mg/day and a maximum dose of 600mg/day. 72,22% of patients received concomitant treatment with benzodiazepines, mood stabilisers, sedative-hypnotic drugs or with another antipsychotic. Antipsychotics used in combination with clozapine were both oral (risperidone, amisulpride, quetiapine, aripiprazole) and long-acting injectable (aripiprazole, risperidone, paliperidone, flupentixol decanoate).

**Conclusions:**

Clozapine remains the drug of choice in treatment-resistant schizophrenia even after 10 years, but its mode of administration has changed over time. While the doses of clozapine used have decreased, the percentage of patients receiving concomitant treatment has doubled.Although some side effects of clozapine are dose-dependent, lowering doses and combining with other adjuvant treatment is not always a better option, as polypragmacy and possible adverse effects combined can lead to reduced adherence.The decision to increase the dose of clozapine or to use concomitant (combination) treatment depends on individual factors, including the patient’s clinical condition, response to treatment, and the assessment of potential risks and benefits.

**Disclosure of Interest:**

None Declared

